# Transcranial Doppler Echography Measurement in Hemodialysis Patients: The Potential Role of Angiotensin II Receptor Blockades on Cerebrovascular Circulation

**DOI:** 10.3390/jcm12062295

**Published:** 2023-03-15

**Authors:** Kyoko Maesato, Shuzo Kobayashi, Takayasu Ohtake, Yasuhiro Mochida, Kunihiro Ishioka, Machiko Oka, Hidekazu Moriya, Sumi Hidaka

**Affiliations:** Department of Kidney and Transplant Center, Shonan Kamakura General Hospital, 1370-1 Okamoto, Kamakura 247-8533, Kanagawa, Japan

**Keywords:** angiotensin II receptor blockade, hemodialysis, intracranial doppler echography, cerebral artery velocity

## Abstract

Background: Although hemodialysis (HD) patients have an elevated risk of strokes, there are few reports about transcranial doppler (TCD) echography measurements. It is well-known that angiotensin II receptor blockades (ARBs) protect against cardiovascular complications. In this study, we measured intracranial artery (ICA) velocity using TCD echography and studied the associated factors with its velocity in HD patients by a comparison with or without ARBs. Methods: We conducted a cross-sectional study in a single hospital. We included 61 patients who had measurable ICA velocity by TCD echography. Among them, the ARB usage group consisted of 22 subjects, whilst the non-ARB usage group consisted of 39 subjects. Results: Patients in the ARB (+) and ARB (-) groups did not show any difference in basic characteristics. ICA blood flow velocity in all intracranial arteries tended to show greater values in the ARB group than those in the non-ARB group. Particularly, blood velocity in the middle cerebral artery (MCA) (maximal flow velocity) statistically increased in the ARB group, respectively. In a univariate analysis, MCA maximum velocity was significantly associated with ARB usage (*p* = 0.011) and low hematocrit levels (*p* = 0.045). The multivariate analysis chose only ARB usage as an independent factor associated with left MCA maximum velocity (*p* = 0.022). Conclusions: We showed that dialysis patients with ARBs have significantly higher ICA blood velocity. ARBs might have a potential benefit for maintaining ICA blood flow in HD patients.

## 1. Introduction

It is well-known that there is a higher risk of cardiovascular and/or cerebrovascular complications in HD patients than in the general population, although the cerebrovascular mortality rate in HD patients has decreased every year in Japan, being 5.5% in 2007 in contrast to 10.8% in 1990 [1].

It should be noted that there are many reports concerning the vasoprotective effects of the renin–angiotensin aldosterone blockade. Reports from the HOPE study [2] and LIFE study [3] demonstrated that angiotensin-converting enzyme inhibitors (ACE-I) or angiotensin II type 1 (AT1) receptor blockades (ARBs) could effectively decrease the incidence of strokes in patients at risk. Moreover, there are a few reports about intima–media thickness (IMT) regression with candesartan [4]. Another report showed the anti-inflammatory and neuroprotective effect of ARBs in a brain ischemia–reperfusion model [5].

Transcranial Doppler (TCD) echography is a useful tool to assess intracranial vascular disease in the general population, especially high-risk patients. However, to our knowledge, little information is available regarding TCD measurements of HD patients.

Therefore, in this study, we measured intracranial artery (ICA) blood flow using TCD echography in HD patients and compared the data with the reference data of ICA velocity obtained from the general population. Furthermore, we evaluated the effectiveness of ARBs on ICA velocity.

## 2. Materials and Methods

### 2.1. Patients

**Figure 1:**
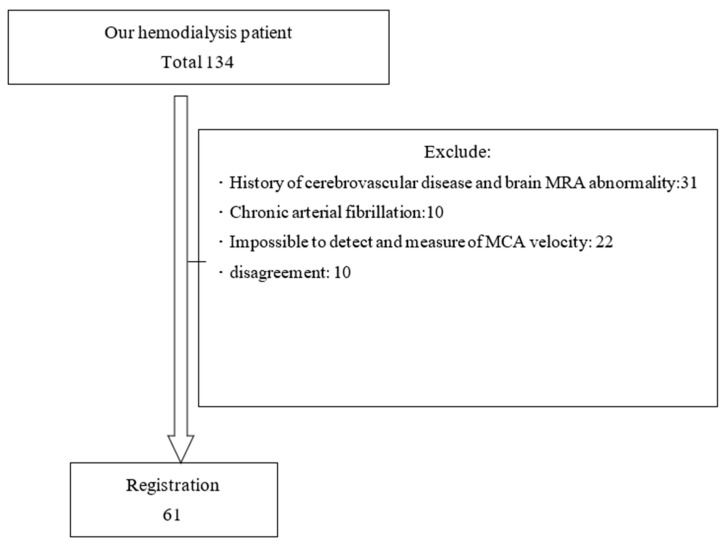
Patient registration.

We performed this cross-sectional study at a single HD center in our hospital (n = 134). Patients with maintenance HD for at least a 3 month period were eligible for entry into the study. The exclusion criteria were as follows: (1) a history of cerebrovascular disease and brain MRA abnormality; (2) a history of chronic atrial fibrillation; (3) failed ICA blood flow measurement by TCD echography due to a narrow temporal bone window, creating an inability to detect arterial signals; and (4) disagreed to be examined by TCD echography (Figure 1). All patients had AVF as a vascular access and none used CVC. We examined the univariate association between ICA blood flow and several factors. ARB usage was adopted in the process; thus, we divided our patients into two groups, with a comparison according to ARB usage with respect to MCA velocity. The ARB usage group consisted of 22 subjects and the non-ARB usage group consisted of 39 subjects.

All subjects gave informed consent and the ethical committee of our hospital approved the present study (approval number: TGE01984-024).

### 2.2. Method

We measured ICA velocity with TCD echography on non-HD days. This method can measure the cerebral vessel flow velocity (maximal flow velocity (Vmax) = peak systolic flow velocity; minimal flow velocity (Vmin) = end diastolic flow velocity). Using temporal, occipital acoustic windows, we measured the flow velocity of the ICA as follows: bilateral middle cerebral artery (MCA); bilateral vertebral artery (VA); and basilar artery (BA). The mean flow velocity (Vmean) was calculated as Vmin + 1/3(Vmax − Vmin). The pulsatility index (PI) was calculated as (Vmax − Vmin)/Vmean. The resistive index (RI) was calculated as (Vmax − Vmin)/Vmax. As the diameter of cerebral arteries is fixed, the PI and RI are influenced by peripheral small artery resistance. We calculated the Vmean, PI and RI for every intracranial artery and compared them in two groups. Due to a few reports showing a laterality of intracranial artery flow velocity, especially during cognitive tasks [6], we compared these data separately.

On the same day, we examined both carotid arteries using a 7.5 MHz linear array transducer with high-resolution B-mode echography (Aloka, Tokyo, Japan). The carotid arteries were examined bilaterally in the areas of the common carotid arteries (1 cm proximal to the dilatation of the carotid bulb), carotid bifurcation (1 cm proximal to the flow divider) and the internal carotid arteries (1 cm distal to the flow divider), according to the method reported by Kobayashi et al. [7]. Intima–media thickness (IMT) was defined as the distance between the leading edge of the lumen–intima echography of the near wall and the leading edge of media–adventitia echography. We then measured the maximum and minimum flow velocity (CCA Vmax) of the common carotid arteries. Two well-trained sonographers performed these measurements. To enhance the reproducibility of the measurements, they used a standardized complementary interrogation.

We calculated the body mass index (BMI) on the same day using the following formula: BMI = body weight (kg)/(height (m))^2^.

Blood samples were drawn from the arterial site of the arteriovenous fistula at the beginning of a dialysis session on a dialysis day. We measured serum calcium, inorganic phosphate (IP), hemoglobin (Hb), hematocrit (Hct), total protein (TP), albumin (Alb), total cholesterol (T-Cho), triglyceride (TG), high-density lipoprotein cholesterol (HDL-C), low-density lipoprotein (LDL-C), high-sensitive C-reactive protein (hsCRP), β_2_-microglobulin (β_2_MG) and plasma fibrinogen levels. We calculated corrected calcium using the following formula: estimated calcium concentration = serum calcium (mg/dL) + (4-serum albumin concentration). As hsCRP was not normally distributed, we used logarithmically transformed hsCRP.

We measured the blood pressure at the beginning of each HD session on the upper arm of the non-venous arterial fistula side.

### 2.3. Statistical Analysis

The continuous data were expressed as the mean ± SD. According to an initial result that concluded ARB usage was significantly associated with ICA velocity, we divided our patients into two groups, ARB use or not, and compared them using the Student’s *t*-test. A univariate regression analysis was performed to assess the predictive factors for ICA velocity. Multiple linear regression analyses were performed to examine the relationship between ICA velocity and the significant factors from the univariate regression analysis. A value of *p* < 0.05 was considered to be statistically significant. All statistical analyses were performed using SPSS version 11.0 (Tokyo, Japan).

## 3. Results

### 3.1. Patient Characteristics

From 134 patients, we included 61 (44 male; 17 female) patients in this study. Table 1 shows the basic characteristics of the patients. Of the patients, 37 (60%) took a form of hypertensive. The numbers of patients given ARBs, calcium channel blockers, angiotensin-converting enzyme inhibitors (ACE-Is), αβ-blockers, β-blockers or α-blockers were 22, 34, 5, 17, 2 and 8, respectively. Of the 5 patients taking ACE-Is, 4 were also taking an ARB. This study was conducted when calcimimetics were not available. A total of 25 patients (41%) were taking vitamin D.

The patients used the following various types of ARB: losartan, 14 patients; candesartan, 1 patient; valsartan, 2 patients; telmisartan, 3 patients; and telmisartan plus losartan, 2 patients. There was no difference in basic characteristics between patients with ARBs and without ARBs (Table 2). We used Hct instead of Hb for rheological reasons.

When we compared the ICA blood flow velocity with that of the non-HD general population as described by Barntt et al. [8] and Krejza et al. [9], the HD patients showed comparable figures (Table 3).

### 3.2. Doppler Echography Measurements

The validities of the TCD measurements in this study via intra- and inter-observer differences were 5.4% and 4.3%, respectively.

The TCA Doppler measurements showed significantly higher maximum flow velocity levels (Vmax) in MCA in the ARB group compared with those in the non-ARB group (Table 4). Bilateral IMT tended to be smaller in the ARB group, although it was not statistically significant in the levels.

For each of the measurements of cerebral arterial blood flow obtained by TCD echography and for each factor, only the left MCA Vmax was significantly correlated with ARB usage (Table 4). In the univariate regression analysis, ARB usage (r = 0.443; *p* = 0.011) and low Hct levels (r = −0.357; *p* = 0.045) were found to be statistically significant factors associated with left MCA Vmax (Table 5).

The multivariate regression analysis chose only ARB usage as an independent association factor (Table 6).

## 4. Discussion

The present study showed statistically significant higher MCA blood flow velocity in patients with ARB usage than without ARB usage in HD patients. Although statistically not significant, the patients with ARBs tended to have a higher velocity in all intracranial arteries and lower IMT in carotid arteries than the patients without ARBs. In the univariate and multivariate regression analyses, ARB usage alone was an independent factor for high blood velocity of MCA. Our results could mean that ARBs also have a vasoprotective effect on intracranial arteries in HD patients, similar to other reports [2,3] in the general population.

The brain has both angiotensin 1 (AT1) and angiotensin 2 (AT2) receptor subtypes. The AT1:AT2 receptor ratio varies substantially with certain brain areas or nuclei, but in most locations, the AT1 receptor is predominant. The classical actions of angiotensin II in the brain include short- and long-term osmoregulation and the control of blood pressure. Under pathological conditions, the expression of the AT2 receptor is known to increase in brain tissue [5]. There are a few mechanisms regarding preserving brain perfusion by angiotensin II receptor blockade, including: (1) ARBs act directly as cerebral vasodilators [4]; (2) ARBs cause outward remodeling in resistance arteries [4]; (3) anti-inflammatory, anti-hyperplasia, anti-fibrous mechanisms promoting the regeneration of the neuron effect by stimulating the AT2 receptor [5]; (4) reducing the expression of the transcription factors c-fos and c-jun (in vivo) and an anti-apoptosis effect [5]; and (5) AT2 receptor-mediated activation of the transcription factor nuclear factor-κB in Schwann cells, leading to enhanced remyelination [5]. This effectiveness of ARBs not only accelerates the process of tissue repair, but also improves functional recovery [5] in brain tissue. Moreover, ARBs are known to improve dyslipidemia by lowering the serum levels of total cholesterol, LDL cholesterol and lipid peroxides (LPO) [10]. 

In our study, although the ARB usage group showed a higher intracranial flow velocity, there was no difference in the PI and RI between the two groups. The reason is still unknown. However, the influence of ARBs may have been greater on Vmax than Vmin, meaning that there was an effect on peripheral small artery resistance at the cardiac systole.

In a general population study that included 598 patients with minor ischemic strokes or transient ischemic attacks, the mean flow velocity in the MCA was significantly correlated with age, male sex, diabetes, total serum cholesterol and hypertension [11]. Hypertensive patients who did not receive HD treatment showed a lower flow velocity in intracranial arteries than non-hypertensive patients [5]. Age and anemia are also associated factors for ICA blood flow velocity, correlating negatively and positively, respectively, as reported in previous literature [12].

The reason we used the left MCA in this study was that it was easier to obtain various parameter values by pulsed Doppler than from the right MCA. Regarding the left–right difference in cerebral blood flow, it has been reported that there is no association with AVF [13]. One theory as to the reason for the left–right difference in the MCA is that the rotation of the cervical spine may significantly affect vertebral artery blood flow [14], thus leading to changes in the blood flow in the basilar artery [15] and MCA [16]. In addition, an autopsy evaluation of the left–right difference in cerebral arterial flow showed significant cerebral infarction and ventricular atrophy in the left side of the brain. The same article also reported that the left cerebral infarction and ventricular atrophy were caused by overwork due to the presence of language centers in the left cerebral hemisphere and differences in the right and left branches of the carotid arteries in the aortic arch [17]. The TCD echography used in this study did not technically evaluate the arterial diameter and we cannot say whether it supported the report in the above article.

An assessment of obstruction of MCA using TCD echography has already been standardized in the general population. However, to the best of our knowledge, no such data are available for HD patients. Therefore, a standardized assessment of MCA obstructions using TCD echography is still obscure. As it has been reported that asymptomatic lacunar infarction is more prevalent in CKD patients prior to HD induction [18], TCD Doppler measurements may be a valuable tool to detect poor conditions in ICA perfusion and to know whether ARBs may be effective against brain protection in high-risk groups such as CKD patients.

This study had several limitations. This was a single-center, small study. Although we were able to identify the factors significantly associated with the outcome (such as ARB) at a conventional significant threshold of *p* < 0.05, we could not adjust for multiple testing with the sample size. Therefore, based on the current study findings, a confirmatory study with a larger sample size may be warranted. Furthermore, the cross-sectional nature of our observations precluded cause–effect inferences about the links between ICA blood flow velocity and ARBs. In terms of assessing intra-individual variations, there were limitations in terms of the study using Doppler echography. Unlike CT and MRI, TCD is a useful tool that can provide information on the dynamic state of cerebral blood flow, but it requires advanced skills of technologists and there are large disparities among technologists and facilities. For this reason, we decided to conduct this study within one facility. Securing an approach window from the temporal region to observe the middle cerebral artery is particularly difficult with older women and many cases were not available for examination. In addition, because cerebral blood flow is affected by arrhythmia, cardiac output, fluid balance, carotid arteriosclerosis, previous strokes, aging and awareness activity, we chose a cross-sectional research approach on this occasion. In this study, the coefficient of variations for the intra- and inter-observer variations were 5.4% and 4.3%, respectively. On the other hand, for the reasons mentioned above, cerebral blood flow velocity values measured by TCD echography may fluctuate and frequent TCD echography examinations may lead to confusion. Therefore, in the present study, we tested and cross-sectionally evaluated outpatients on non-dialysis days, when the fluid balances were relatively stable. To assess intra-individual variability in the future, TCD echography should be performed regularly under similar conditions and changes over time should be evaluated if the same subjects do not suffer a stroke.

We showed the ICA blood flow in more than fifty HD patients; the strength of the associations of ICA flow with ARBs that appeared from our study form a basis for conducting cohort and intervention studies.

## 5. Conclusions

We successfully performed TCD Doppler measurements for ICA blood flow velocity despite severe atherosclerotic conditions in HD patients. Moreover, ARBs may have beneficial effects on brain perfusion, particularly in MCA, in HD patients.

## Figures and Tables

**Table 1:** Baseline patient characteristics.

	N = 61
Gender (male/female)	44/17
Age (years)	67.8 ± 11.4
DM (+%)	27%
Smoking (+%)	49%
BMI	21.1 ± 2.9
HD vintage (m)	94.2 ± 58.6
sBP (mmHg)	149.7 ± 15.8
dBP (mmHg)	82.7 ± 11.1
Pulse pressure (mmHg)	66.8 ± 12.5
α-Blocker (n)	8 (18%)
β-Blocker (n)	2 (3%)
αβ-Blocker (n)	17 (28%)
CCB (n)	34 (56%)
ACE-I (n)	5 (8%)
ARB (n)	22 (36%)
Vitamin D (n)	25 (41%)

BMI: body mass index; sBP: systolic blood pressure; dBP: diastolic blood pressure; ARB: angiotensin II receptor blockade; CCB: calcium channel blocker; ACE-I: angiotensin-converting enzyme inhibitor.

**Table 2:** Data according to the presence or absence of ARBs.

	ARB (+) N = 22	ARB (-) N = 39	*p*-Value
Gender (M/F)	17/5	27/12	0.509
Age (years)	64.3 ± 10.2	69.8 ± 11.7	0.070
DM (%)	27	28	0.939
Smoking (%)	55	44	0.419
BMI	20.7 ± 2.9	21.3 ± 3.0	0.428
HD vintage (months)	100.9 ± 54.6	90.4 ± 61.1	0.506
sBP (mmHg)	154.7 ± 10.9	146.6 ± 17.5	0.061
dBP (mmHg)	84.3 ± 10.2	81.7 ± 11.7	0.406
Pulse pressure (mmHg)	70.2 ± 12.4	64.7 ± 12.2	0.112
Ca (mg/dL)	9.3 ± 1.1	8.9 ± 0.9	0.139
IP (mg/dL)	6.1 ± 1.2	5.9 ± 1.3	0.563
Ca × IP	56.3 ± 10.4	52.8 ± 13.1	0.277
Intact PTH (ng/dL)	350.5 ± 198.9	270.6 ± 211.4	0.151
Hct (%)	30.9 ± 4.0	32.6 ± 4.5	0.154
Alb (g/dL)	3.7 ± 0.3	3.6 ± 0.4	0.624
β_2_MG (mg/L)	30.2 ± 6.7	28.5 ± 6.0	0.319
Log hsCRP	2.35 ± 1.67	1.85 ± 1.33	0.205
Fibrinogen (mg/dL)	300 ± 77.3	336.2 ± 86.2	0.107
CCB (%)	63.6	51.3	0.419
αβ-Blocker (%)	27.3	28.2	0.936

ARB: angiotensin II receptor blockade; DM: diabetes mellitus; BMI: body mass index; HD: hemodialysis; BP: blood pressure; Ca: calcium; IP: inorganic phosphate; iPTH: intact parathyroid hormone; Hct: hematocrit; Alb: albumin; β_2_MG: β_2_-microglobulin; hsCRP: high-sensitive C-reactive protein; CCB: calcium channel blocker.

**Table 3:** Transcranial Doppler measurements of our HD patients and normal references [8,9].

		Our HD Patients N = 61	Normal N = 335 (Age > 60)
Rt MCA	Vmax (cm/s)	90.5 ± 27.4	92
	Vmin (cm/s)	34.1 ± 11.1	37
	Vmean (cm/s)	51.6 ± 16.6	59
	PI	1.19 ± 0.5	0.96 ± 0.17
	RI	0.62 ± 0.1	0.60 ± 0.06
Lt MCA	Vmax (cm/s)	86.0 ± 31.1	92
	Vmin (cm/s)	31.2 ± 11.0	37
	Vmean (cm/s)	49.4 ± 16.2	59
	PI	1.10 ± 0.27	0.96 ± 0.17
	RI	0.60 ± 0.08	0.60 ± 0.06
Rt VA	Vmax (cm/s)	51.2 ± 18.5	50.9 ± 18.7
	Vmin (cm/s)	20.1 ± 6.5	21.2 ± 9.2
	Vmean (cm/s)	30.4 ± 9.9	30.5 ± 12.4
	PI	1.01 ± 0.24	
	RI	0.60 ± 0.09	
Lt VA	Vmax (cm/s)	48.6 ± 19.6	50.9 ± 18.7
	Vmin (cm/s)	20.0 ± 8.7	21.2 ± 9.2
	Vmean (cm/s)	29.5 ± 12.1	30.5 ± 12.4
	PI	0.98 ± 0.21	
	RI	0.59 ± 0.07	
BA	Vmax (cm/s)	59.5 ± 21.6	62
	Vmin (cm/s)	23.0 ± 8.4	26
	Vmean (cm/s)	35.2 ± 12.3	40
	PI	1.04 ± 0.23	0.94 ± 0.16
	RI	0.61 ± 0.08	0.60 ± 0.09

Vmax: max velocity; Vmin: minimal velocity; Vmean: mean velocity; PI: pulsatility index; RI: resistive index; Rt: right; Lt: left; MCA: middle cerebral artery; VA: vertebral artery; BA: basilar artery.

**Table 4:** ICD measurements (maximal velocity) in HD patients.

	ARB (+) N = 22	ARB (-) N = 39	*p*-Value
Rt MCA (cm/s)	103.0 ± 30.3	84.5 ± 24.4	0.065
Lt MCA (cm/s)	105.7 ± 38.2	75.9 ± 23.3	0.011
Rt VA (cm/s)	56.1 ± 21.8	46.9 ± 15.8	0.068
Lt VA (cm/s)	53.5 ± 25.0	44.8 ± 14.0	0.101
BA (cm/s)	65.6 ± 28.3	55.7 ± 16.4	0.142
Rt CCA max IMT (mm)	0.8 ± 0.2	0.9 ± 0.3	0.102
Lt CCA max IMT (mm)	0.8 ± 0.2	0.9 ± 0.3	0.084

MCA: middle cerebral artery; VA: vertebral artery; BA: basilar artery; CCA: common carotid artery; IMT: intima–media thickness.

**Table 5:** Univariate regression analysis for maximal velocity of left MCA.

	r	*p*-Value
Age	0.110	0.563
HD vintage	0.273	0.145
DM+/−	0.094	0.622
Smoking+/−	0.260	0.165
Hct	−0.357	0.045
Albumin	0.233	0.216
β₂MG	0.043	0.824
Log hsCRP	0.043	0.824
Fibrinogen	0.050	0.183
sBP	0.265	0.181
dBP	0.190	0.343
Pulse pressure	0.332	0.078
ARB	0.443	0.011
CCB	0.227	0.227

HD: hemodialysis; DM: diabetes mellitus; Hct: hematocrit; β_2_MG: β_2_-microglobulin; hsCRP: high-sensitive CRP; sBP: systolic blood pressure; dBP: diastolic blood pressure; ARB: angiotensin II receptor blockade; CCB: calcium channel blocker.

**Table 6:** Stepwise multiple regression analysis for independent determinants of maximal velocity of left MCA.

	95% CI	β	*p*-Value
ARB usage	3.961–48.464	0.389	0.022
Hct	−4.210–0.37	−0.282	0.091

ARB: angiotensin II receptor blockade; Hct: hematocrit.

## Data Availability

The data presented in this study are available on reasonable request. The data are not publicly available because they are the property of Shonan Kamakura General Hospital.
